# Purpura Hæmorrhagica Fulminans

**Published:** 1903-09-12

**Authors:** 


### PURPURA HÆMORRHAGICA FULMINANS.

In all probability it will fall to the lot of but few medical practitioners to be called upon to treat this rare condition, of which, however, several instances have recently been recorded in the medical journals. In the fulminating type of hæmorrhagic purpura the invasion is generally rapid and severe, the bleeding taking place not only into the skin but into the mucous membranes and all the internal organs and tissues. The bleeding from the nose is especially severe, and hæmorrhage from the gums and palate is common. The patient is usually overwhelmed by the hæmorrhages, and death commonly occurs within a few hours to two days. There is almost invariably a fatal termination. The diagnosis, as a rule, is easy, though cases have occurred in which severe internal hæmorrhage has caused death without there being any external evidences of purpura. Generally speaking the sudden and violent onset, with bleeding into every tissue and organ in the body, is sufficiently characteristic. With regard to the etiology of the condition numerous theories have been advanced and many various micro-organisms have been isolated from the blood and tissues of affected patients, but so far no definite conclusions have been arrived at. With regard to treatment, absolute rest is indicated, with the avoidance as far as possible of even the slightest injury. Beyond this innumerable drugs have been tried, including the various hæmostatics, but the success has been infinitesimal. However, Dr. H. G. MacAdam[1] reports a typical case which came under his care, and which recovered under treatment, which consisted of the internal administration of adrenalin solution. The patient was a girl child, five years old, who had been perfectly healthy up to the onset of the attack in question. On January 9th the child was drowsy and heavy in the morning, in the afternoon three "bruises" appeared on her forehead. As no history of injury was forthcoming the mother sought medical advice and the child was first seen by Dr. MacAdam on the following day at 11 A.M. At that time she was playing about and seemed pretty well. Being stripped, she was seen to be covered with petechiæ, and there were some half-dozen ecchymotic spots on the body. At 11.30 A.M. Dr. MacAdam was sent for hurriedly, and, he being unable to attend, another physician went in his place. The child was found almost moribund, unconscious, ashy-white, and bleeding from the nose, stomach, lungs, bowels and kidneys. The pulse was imperceptible. The child had vomited a large quantity of blood. Local applications of adrenalin chloride 1–1,000 were made to the nose but no other treatment was adopted. Within an hour Dr. MacAdam saw the child, who had continued to vomit blood, and the case seemed hopeless. As a last resource twenty drops of the adrenalin solution were given internally in a tablespoonful of water, fifteen minutes after another ten drops were given, and the child began to show signs of improvement. Subsequently ten minims of adrenalin solution were given every three hours and continued till January 31st. The child steadily improved, and although there were some further hæmorrhages ultimate recovery was complete. In the light of this case Dr. MacAdam is of opinion that in adrenal-gland preparation we have a certain and reliable constitutional hæmostatic.

[1] Medical Record, August 22.

